# Health workforce strategies in response to major health events: a rapid scoping review with lessons learned for the response to the COVID-19 pandemic

**DOI:** 10.1186/s12960-021-00698-6

**Published:** 2021-12-20

**Authors:** Alison Coates, Asli-Oubah Fuad, Amanda Hodgson, Ivy Lynn Bourgeault

**Affiliations:** 1 Telfer School of Management, University of Ottawa, Ottawa, Canada; 2 Faculty of Medicine, University of Ottawa, Ottawa, Canada; 3 University of Ottawa Library, Ottawa, Canada; 4 Present Address: Canadian Agency for Drugs and Technologies in Health (CADTH), Ottawa, Canada; 5 School of Sociological and Anthropological Studies, University of Ottawa & Lead, Canadian Health Workforce Network, Ottawa, Canada

**Keywords:** Health workforce, COVID-19, Emergency response, Pandemic response, Scope of practice, Surge capacity, Health human resources, Coronavirus

## Abstract

**Background:**

The early weeks of the COVID-19 pandemic brought multiple concurrent threats—high patient volume and acuity and, simultaneously, increased risk to health workers. Healthcare managers and decision-makers needed to identify strategies to mitigate these adverse conditions. This paper reports on the health workforce strategies implemented in relation to past large-scale emergencies (including natural disasters, extreme weather events, and infectious disease outbreaks).

**Methods:**

We conducted a rapid scoping review of health workforce responses to natural disasters, extreme weather events, and infectious disease outbreaks reported in the literature between January 2000 and April 2020. The 3582 individual results were screened to include articles which described surge responses to past emergencies for which an evaluative component was included in the report. A total of 37 articles were included in our analysis.

**Results:**

The reviewed literature describes challenges related to increased demand for health services and a simultaneous decrease in the availability of the workforce. Many articles also described impacts on infrastructure that hindered emergency response. These challenges aligned well with those faced during the early days of the COVID-19 pandemic. In the published literature, the workforce strategies that were described aimed either to increase the numbers of health workers in a given area, to increase the flexibility of the health workforce to meet needs in new ways, or to support and sustain health workers in practice. Workforce responses addressed all types and cadres of health workers and were executed in a wide range of settings. We additionally report on the barriers and facilitators of workforce strategies reported in the literature reviewed. The strategies that were reported in the literature aligned closely with our COVID-specific conceptual framework of workforce capacity levers, suggesting that our framework may have heuristic value across many types of health disasters.

**Conclusions:**

This research highlights a key deficiency with the existing literature on workforce responses to emergencies: most papers lack substantive evaluation of the strategies implemented. Future research on health workforce capacity interventions should include robust evaluation of impact and effectiveness.

**Supplementary Information:**

The online version contains supplementary material available at 10.1186/s12960-021-00698-6.

## Background

The early weeks of the COVID-19 pandemic brought great uncertainty, anxiety and, in some cases, panic to health systems across the globe. As clinical and experiential stories were shared from Hunan, China [1], to Lombardy, Italy [2], and to New York City, USA [3], health systems stakeholders began to reckon with the scale and complexity of the emerging crisis. Facing multiple concurrent threats—high patient volume and acuity and, simultaneously, increased risk to healthcare workers—healthcare managers and decisionmakers quickly rallied to anticipate the pressures impacting the health workforce and to identify strategies to mitigate adverse conditions [4, 5]. COVID-19 created an unprecedented need for innovation to respond to patient, population, and health worker needs.

**Fig. 1:**
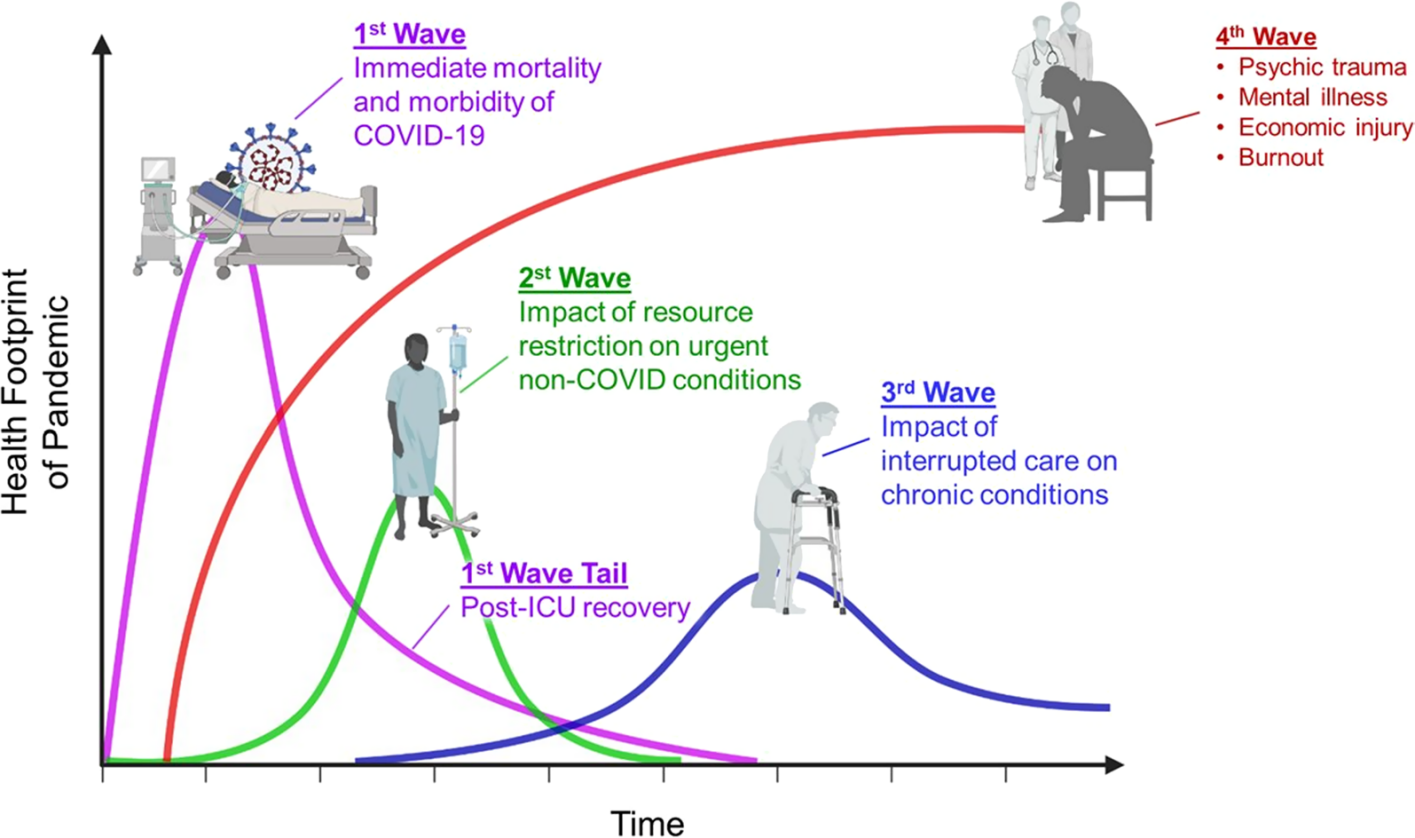
Four Waves of a Pandemic, Dr. Victor Tseng, ICU/Critical Care Physician based in Atlanta; reprinted with permission

It was recognized early on that the COVID-19 pandemic would likely have short, medium and long-term impacts coming in four waves (Fig. 1) [6]. (*Here, the term “wave” refers to the phases of impact of the pandemic, which is different from how “wave” has come to be known as a period of increased viral spread.*) The first wave of impact, depicted in purple, involves the immediate and acute response to a pandemic-like COVID-19, including the active treatment of severely ill patients and their post-acute recovery and a tail of post-intensive care unit (ICU) recovery and readmissions. A *second* non-COVID wave depicts the backlog in other urgent, but non-COVID conditions. A *third* wave depicts the impact of interrupted care on chronic conditions. The backdrop to each of these waves is a *fourth* wave comprising economic injury, burnout and psychological trauma within the broader population as well as the health workforce itself.

In the context of the first wave of an acute patient surge, researchers at the Canadian Health Workforce Network (CHWN) were approached by Healthcare Excellence Canada^1^ to undertake an urgent national and international scan of promising strategies that could help to address COVID-19-related workforce challenges. Our approach took a broad view of the health workforce (including regulated and unregulated health professionals in a diversity of settings) and was designed to include a wide variety of potential solutions.

Our approach to gathering strategies and promising practices took two complementary paths: performing an environmental scan of the Canadian and international workforce strategies developed to respond to COVID-related challenges (publication forthcoming), and, concurrently, undertaking a rapid scoping review of the published literature on health workforce strategies reported in relation to past health emergencies (including natural disasters, extreme weather events, and infectious disease outbreaks). This latter project is reported here.

### Conceptual framework

COVID-19 presented three key synergistic pressures to the health system. First, the severity and clinical course of the disease placed unprecedented *acuity demand* on the health system’s capacity and its workforce which simultaneously needed to maintain essential non-COVID care. Second, the epidemiology and infectiousness of COVID-19 contributed to a *volume demand* for acute care services. The third pressure involved *attrition* in the supply of health workers due to illness, exposure/quarantine, family illness, lack of childcare, and fear (and the related effects on mental health). As a result, health systems were facing a situation of high need and an unstable and likely diminishing capacity to meet that need.

**Fig. 2:**
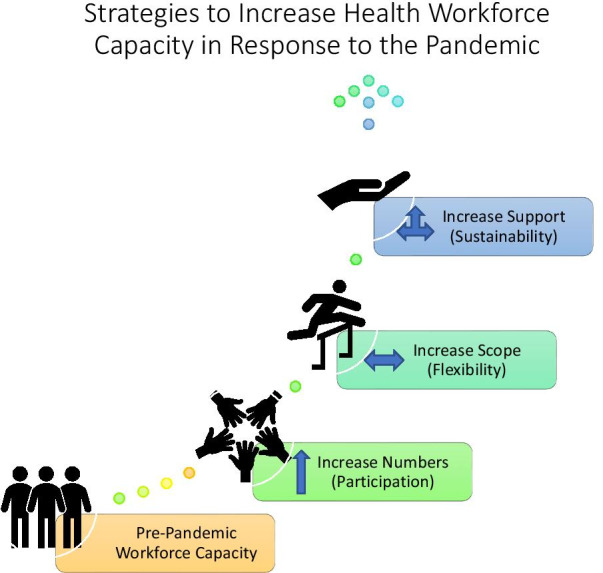
Conceptual framework of strategies to increase health workforce capacity in response to acute crises

In responding to the triple threat of pressures, we identified three types of necessary responses to shore up the pre-pandemic health workforce (Fig. 2): (1) addressing the *volume demand* requires strategies focused on *increasing numbers* of health workers participating in the provision of care (both COVID-19 and non-COVID-19 related); (2) addressing a *skills demand* requires strategies focused on *increasing flexibility* of the workforce to increase among other things the scope within and between cadres of health workers; and (3) addressing the need for *sustained healthcare worker availability* requires strategies focused on *increasing support* of workers in practice.

### Why conduct a scoping review

As the health systems across the globe scrambled to implement strategies to boost the health workforce in areas of acute need, we recognized that few of these initiatives would have any outcome data yet related to their feasibility, impact, or their risk to the health system (including risks to patients, providers, and organizations). We thus looked to the published literature to understand whether workforce strategies devised to respond to previous acute health system crises could provide an evidentiary foundation for the development of COVID-19-related responses. Many of the health system pressures that were anticipated in the early days of the pandemic resembled those seen in natural disasters, extreme weather events, and in previous infectious disease outbreaks. For example, the aforementioned types of emergencies all tend to involve early surges of injured or ill patients, often experiencing a reduction in health and local infrastructure, and with the disaster itself impacting local health workers in addition to the community members. Our objective for the scoping review portion of our project was to identify health workforce innovations that have been developed or implemented in the face of historical large-scale health events, such as an infectious disease outbreaks or natural disasters, and to identify the potential impact, risks, barriers and facilitators related to each. As a secondary objective, we wanted to apply our conceptual model of pressures and response strategies as a coding frame to support its use in the concurrent environmental scan of COVID-19-related workforce pressures and related responses.

## Methods

The aim of this project was to identify workforce strategies designed to strengthen the health workforce in face of large-scale health events such as infectious disease outbreaks or natural disasters and to understand the impact, risks, barriers and facilitators related to such responses. Rapid reviews are typically conducted when a scan of available evidence is needed within a short timeframe. Our team was contracted to respond acutely to the COVID-19 pandemic in progress and to provide meaningful information within 8–12 weeks; thus, a rapid review methodology was deemed appropriate [7]. To assess the breadth of literature available on the topic of urgently implemented health workforce innovations, a scoping review approach was undertaken [8]. We follow the scoping review framework described by Arksey and O’Malley [8] with certain accommodations made to accommodate for the compressed timeframe, consistent with rapid review methods found in the literature [9].

A peer reviewed search strategy [10] was constructed and implemented by a research librarian [AH] in OVID MEDLINE, Embase, and EBSCO CINAHL databases. The full search strategy can be found in Additional file 1. The search was limited to English or French language articles published from January 1, 2000 to April 23, 2020. Duplicate entries were removed to yield 3582 unique results. Prior to beginning the screening and analysis phases of the study, a rapid scoping review protocol was developed and registered with OSF [11].

Two stages of screening were performed to identify only workforce strategies targeting the initial wave of response to the emergency, and where some degree of evaluation was reported in the article. Two team members [AC & AF] screened the titles and abstracts using Covidence software. Following successive pilot tests of the screening criteria performed in duplicate and reaching at least 75% concordance, this initial screening phase was performed in singlicate. Abstracts were included if they mentioned a major medical event (disease outbreak or natural disaster) either generally or specifically, if it referred to at least one type of health worker, and if it referred to workforce changes that would increase the supply, flexibility, or availability of the health workforce. Articles referring only to war, armed conflict, or mass casualty events were excluded, as were studies, where health workers were the subjects but not the objects of the research (for example, if nurses were recruited as subjects and interviewed about a topic, such as patient needs during pandemics). Articles that presented a personal narrative without explicitly considering the outcomes of the effort in some way were also excluded from analysis.

**Fig. 3:**
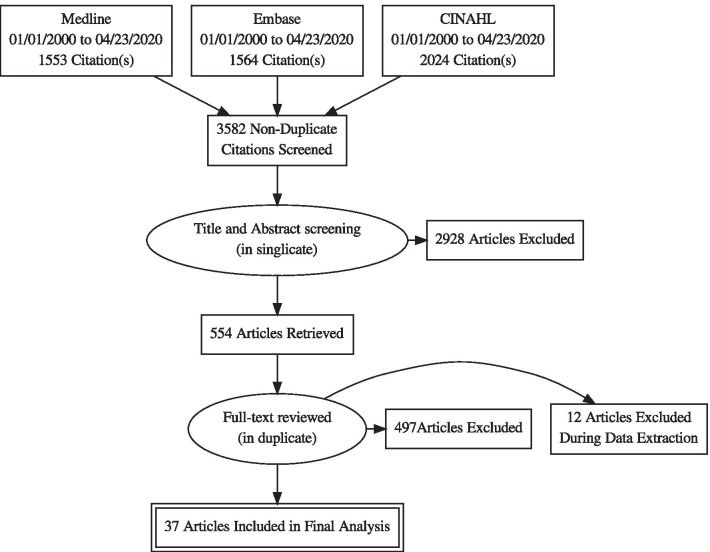
PRISMA flow diagram

Of the 654 articles which entered the full text screening phase, 100 were excluded due to the inability to access a copy for review within the time constraints of the project. The remaining 554 full texts were read by two team members and screened in duplicate. Full text articles were included if they provided some degree of evaluation of the workforce strategy (for example, impact or outcome assessments or “lessons learned”) but excluded if they merely described and/or reflected on a response to an emergency. Purely theoretical papers and simulation studies were also excluded. Articles related to the COVID-19 pandemic were included in this phase if they met screening criteria though these were subsequently excluded to focus our evaluation on pre-COVID-19 knowledge. Figure 3 diagrams the search and screening process undertaken.

**Fig. 4:**
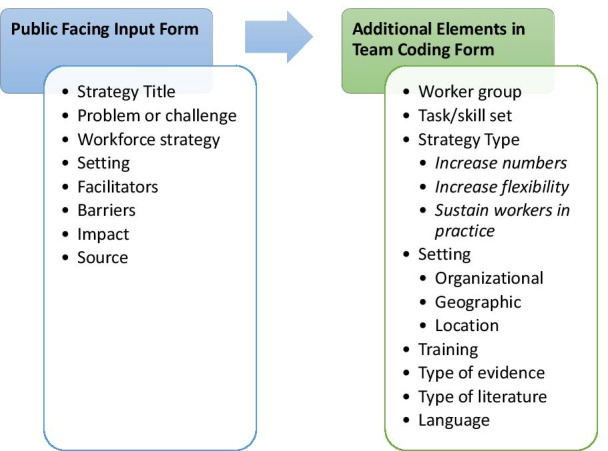
Coding scheme for article extraction

Data were charted in singlicate by a member of the research team [AC] using a Microsoft Excel spreadsheet following a standardized coding scheme (Fig. 4). Our analytical coding frame was developed based on our conceptual model specifically designed to identify and capture workforce strategies for responding to COVID-19. In this scoping review, we inductively coded the included articles with respect to the workforce-related challenges that they described. The challenges described in the extant literature aligned well with the coding frame devised for COVID-19, which supported our decision to code the workforce strategies deductively into the conceptual model’s three types of workforce responses (increasing numbers, increasing flexibility, and increasing support).

From each article we extracted a brief textual description of the setting, the intervention, the problem or challenge that the strategy addressed, whether training was involved, any barriers and facilitators to implementation, whether and how the innovation was assessed, and the outcome or impact of the innovation. Team members charted the health care worker group(s) involved in each workforce strategy, any specific tasks or skills referenced, and the organizational and geographic settings. We inductively coded the types of challenges described in each of the articles and deductively coded the workforce responses according to the conceptual framework established a priori and described above. Finally, we inductively coded the articles for factors that enabled or hindered the workforce strategies described.

## Results

The vast majority of articles that were excluded during screening were anecdotal personal accounts of a health care provider’s response to a disaster, where no evaluative component was reported. Of the 37 papers included in the analysis, 9 related to infectious disease outbreaks [H1N1 influenza, Ebola virus, Severe Acute Respiratory Syndrome(SARS)] (Table 1) [12–20]; 19 related to extreme weather events (hurricanes, typhoons, severe storms) (Table 2) [21–39], and ten related to natural disasters (earthquakes, tsunamis) (Table 3) [40–49]. Twenty-four of the papers (65%) were observational/descriptive papers, seven (19%) were qualitative or mixed-methods case studies, two (5%) reported on survey data, two (5%) on qualitative studies, two (5%) were systematic reviews, and one (2.5%) was a quantitative study. The strategies were most commonly evaluated and reported as “lessons learned” (20/37, 54%) or through impact data, such as numbers of patients and procedures addressed within the strategy (19/37, 51%), or numbers and types of workers involved in the strategy (12/37, 32%). Other evaluative elements reported included challenges encountered during the strategy implementation (8/37, 22%), enablers of the strategy (5/37, 14%), and costs of implementation (2/37, 5%).

**Table 1:** Articles related to infectious disease outbreaks

Infectious disease outbreaks							
First author (year) (citation)	Crisis (year)	Types of challenges	Types of strategies	Provider groups	Setting	Type of article	Types of evaluations
Booth (2005) [12]	SARS (2003)	Mass casualty/patient surge, damaged/reduced/insufficient facilities, loss of workforce	Increase numbers: broader scope of practice Increase flexibility: telehealth/virtual care Increase support: mental health counseling for front line workers	Physicians, nurses	Critical care, hospitals	Observational–descriptive	Lessons learned
Chaple (2017) [13]	Ebola epidemic (2013–2014)	Mass casualty/patient surge, damaged/reduced/insufficient facilities, loss of workforce	Increase numbers: solidarity staffing (e.g., deployments to/from other jurisdictions)	Physicians, nurses	Hospitals	Observational–descriptive	Data about patients/procedures, data about workforce
Charney (2012) [14]	H1N1 influenza (2009)	Mass casualty/patient surge	Increase numbers: cross-sector staff deployments Increase flexibility: cross-sector deployment	Physicians, nurses	Emergency medical services, hospitals	Observational–descriptive	Data about patients/procedures, data about costs
Chin (2004) [15]	SARS (2003)	Excess staff within unit	Increase flexibility: alternative deployments for health workers whose normal duties are temporarily suspended	Pharmacists	Hospitals	Observational–descriptive	Lessons learned
Chou (2010) [16]	SARS (2003)	Mass casualty/patient surge, loss of workforce	Increase numbers: back-up solutions for absenteeism, dedicated hospital Increase flexibility: rapid upskilling/reskilling existing and available workers (e.g., laid off), alternative deployments for health workers whose normal duties are temporarily suspended, expanded roles Increase support: Mental health services, Housing for front-line workers	Nurses, military health workers	Critical care, hospitals	Observational–descriptive	Lessons learned
Considine (2011) [17]	H1N1 influenza (2009)	Mass casualty/patient surge, loss of workforce	Increase flexibility: alternative deployments for health workers with underlying health conditions, alternative deployments for health workers whose normal duties are temporarily suspended, cross-sector deployment	Physicians, nurses, students	Emergency medical services, hospitals	Analytical–survey	Data about workforce (redeployment and absenteeism)
Corley (2010) [18]	H1N1 influenza (2009)	Mass casualty/patient surge	Increase numbers: overtime hours Increase flexibility: rapid upskilling/reskilling existing and available workers (e.g., laid off), task shifting/delegation, new roles, expanded roles	Nurses	Critical care, hospitals	Analytical–qualitative	Data about workforce experience
Crawford (2010) [19]	H1N1 influenza (2009)	Mass casualty/patient surge	Increase numbers: cross-sector staff deployments Increase flexibility: task shifting/delegation Increase support: mandatory off duty rotation	Medical laboratory workers	Diagnostic services	Observational–descriptive	Data about patients/procedures, lessons learned
Cruz (2010) [20]	H1N1 influenza (2009)	Mass casualty/patient surge	Increase numbers: Field hospital Increase flexibility: alternative deployments for health workers whose normal duties are temporarily suspended	Physicians, nurses, medical laboratory workers,	Emergency medical services, hospitals	Observational–descriptive	Data about patients/procedures, lessons learned

**Table 2:** Articles related to extreme weather events

Extreme weather events							
First author (year) (citation)	Emergency (year)	Types of challenges	Types of strategies	Provider groups	Organizational setting	Type of article	Types of evaluations
Albahari (2017) [21]	Sudan Floods (2013)	Unmet health and social needs	Increase numbers: Volunteer aid including healthcare and dental care Increase support: training and psychological support for volunteers	Volunteers	Community health services, other	Analytical–qualitative Case Study	Quality of response measured against a framework (Sphere Handbook)
Broz (2009) [22]	Hurricane Katrina (2005)	Unmet health and social needs	Increase numbers: cross-sector staff deployments, auxiliary health clinic	Physicians, nurses, public health workers, students	Community health services, primary health care	Analytical–mixed methods case study	Lessons learned
Buajaroen (2013) [23]	Thailand flooding (2011)	Damaged/reduced/insufficient facilities, unmet health and social needs	Increase numbers: solidarity staffing (e.g., deployments to/from other jurisdictions), cross-sector staff deployments	Physicians, nurses, students, volunteers	Community health services, critical care, emergency medical services, hospitals, primary health care, public health	Analytical–qualitative case study	Data about workforce; data about services; data about cost
Comeau (2014) [24]	Hurricane Ike (2008)	Damaged/reduced/insufficient facilities	Increase flexibility: rapid upskilling/reskilling existing and available workers (e.g., laid off), longer term upskilling/reskilling other workers, alternative deployments for health workers whose normal duties are temporarily suspended, new roles, expanded roles	Physicians, nurse specialists	Critical care, hospitals	Observational–descriptive	Data on patients/procedures (outcomes), enablers
Connelly (2006) [25]	Hurricane Katrina (2005)	Damaged/reduced/insufficient facilities, mass casualty/patient surge, unmet health and social needs	Increase numbers: solidarity staffing (e.g., deployments to/from other jurisdictions) Increase support: Enabling communication with home, immunization services for volunteer medical providers	Physicians, nurses, paramedics	Critical care, emergency medical services, hospitals	Observational–descriptive	Lessons learned
Currier (2006) [26]; Bailey (2008) [27]	Hurricane Katrina (2005)	Unmet health and social needs	Increase numbers: solidarity staffing (e.g., deployments to/from other jurisdictions), cross-sector staff deployments, interjurisdictional mobility, medical volunteerism Increase support: Provision of child care services for front-line workers, gasoline	Physicians, nurses, dental workers, mental health workers, midwives, pharmacists	Primary health care, other	Analytical–multiple method case review	Data about patients/procedures, lessons learned
D'Amore (2005) [28]	Tropical Storm Allison (2001)	Damaged/reduced/insufficient facilities, mass casualty/patient surge, unmet health and social needs	Increase numbers: solidarity staffing (e.g., deployments to/from other jurisdictions), emergency relief field hospital Increase support: Mental health services, Housing for front-line workers	Physicians, nurses, medical imaging workers, medical laboratory workers, mental health workers, military health workers, pharmacists, public health workers	Critical care, diagnostic services, emergency medical services, hospitals, public health, other	Observational–descriptive	Data about patients/procedures, challenges (problems), lessons learned
Deal (2006) [29]	Hurricane Rita (2005)	Unmet health and social needs	Increase numbers: cross-sector staff deployments Increase flexibility: task shifting/delegation	Nurses, community health workers, personal support workers, students, volunteers	Long-term care, other	Observational–descriptive	Challenges/enablers (opportunities), lessons learned
Edwards (2007) [30]	Hurricane Katrina (2005)	Unmet health and social needs	Increase numbers: cross-sector staff deployments, Staffing a triage center Increase flexibility: cross-sector deployment	Physicians, nurses, mental health providers, pharmacists, students, volunteers	Community health services, diagnostic services, emergency medical services, mental health services, primary health care, public health	Observational–descriptive	Data about patients/procedures, data about workforce, lessons learned
Grover (2020) [31]	Hurricane Florence	Unmet health and social needs	Increase flexibility: telehealth/virtual care, task shifting/delegation, expanded roles	Physicians, nurses, paramedics	Community health services, other	Analytical–quantitative	Data about patients/procedures; effectiveness of approach
Klein (2007) [32]	Hurricane Katrina (2005)	Damaged/reduced/insufficient facilities, mass casualty/patient surge, unmet health and social needs	Increase numbers: solidarity staffing (e.g., deployments to/from other jurisdictions), Emergency medical relief, field hospital	Physicians, nurses, paramedics, pharmacy workers	Critical care, diagnostic services, emergency medical services, hospitals, primary health care, public health	Observational–descriptive	Data about workforce, challenges (problems)
Lawlor (2014) [33]	Tropical Cyclone Yasi (2011)	Mass casualty/patient surge	Increase flexibility: alternative deployments for health workers whose normal duties are temporarily suspended Increase support: Provision of child care services for front-line workers	Nurses, volunteers	Hospitals	Analytical–survey	Data about services, perceptions of services
Parak (2019) [34]	Hurricane Maria (2017)	Damaged/reduced/insufficient facilities, mass casualty/patient surge, unmet health and social needs	Increase flexibility: alternative deployments for health workers whose normal duties are temporarily suspended, task shifting/delegation, new roles, cross-sector deployment, expanded roles	Physicians, nurses	Emergency medical services, hospitals	Observational–descriptive	Data on patients/procedures, (enablers) what went well
Read (2016) [35]	Typhoon Haiyan (2013)	Damaged/reduced/insufficient facilities, mass casualty/patient surge, unmet health and social needs	Increase numbers: solidarity staffing (e.g., deployments to/from other jurisdictions), international emergency medical relief, field hospital;	Physicians, nurses, paramedics	Critical care, diagnostic services, emergency medical services, hospitals	Analytical–prospective case study	Data about patients/procedures,
Taylor (2007) [36]	Hurricane Wilma (2005)	Mass casualty/patient surge, unmet health and social needs	Increase numbers: mobile medical vans	Physicians, nurses, pharmacists, public health workers, social workers	Community health services, mental health services, primary health care, other	Observational–descriptive	data about patients/procedures
Waisman (2003) [37]	Hurricane Mitch (1998)	Mass casualty/patient surge, unmet health and social needs, reduced workforce	Increase numbers: solidarity staffing (e.g., deployments to/from other jurisdictions), international emergency relief	Physicians	Emergency medical services, hospitals, primary health care	Observational–descriptive	Data about patients/procedures, enablers/challenges
Weeks (2007) [38]	Hurricane Katrina (2005)	Unmet health and social needs	Increase numbers: cross-sector staff deployments, shelter volunteering Increase support: mental health services	Nurses, mental health providers, social workers, students	Emergency medical services, mental health services, primary health care	Observational–descriptive	Lessons learned
Wyte-Lake (2018) [39]	Superstorm Sandy (2012)	Excess staff within unit	Increase numbers: cross-sector staff deployments Increase flexibility: alternative deployments for health workers whose normal duties are temporarily suspended, cross-sector deployment Increase support: housing for front-line workers, Home support for front-line workers, Transportation for redeployed workers	All hospital workers	Critical care, diagnostic services, emergency medical services, hospitals, mental health services	Analytical–qualitative case study	Lessons learned

**Table 3:** Articles related to natural disasters

Natural disasters							
First author (year)	Emergency (year)	Types of challenges	Types of strategies	Provider groups	Setting	Type of article	Types of evaluations
Amat Camacho (2019) [40]	Nepal earthquake (2015)	Damaged/reduced/insufficient facilities, mass casualty/patient surge	Increase numbers: solidarity staffing (e.g., deployments to/from other jurisdictions)	Physicians, nurses, military health workers	Critical care, emergency medical services, hospitals, public health	Literature review and case study	Data about patients/procedures, data about workforce
Burnweit (2011) [41]	Haiti earthquake (2010)	Damaged/reduced/insufficient facilities, mass casualty/patient surge, unmet health and social needs	Increase numbers: solidarity staffing (e.g., deployments to/from other jurisdictions), International Emergency Medical Relief, Field hospital Increase support: housing for front-line workers	Physicians, nurses, pharmacists, social workers, medical imaging workers	Critical care, diagnostic services, emergency medical services, hospitals	Observational–descriptive	Data about patients/procedures, Data about workforce; Challenges (pitfalls)
Catlett (2011) [42]	Haiti earthquake (2010)	Damaged/reduced/insufficient facilities, mass casualty/patient surge, unmet health and social needs	Increase numbers: solidarity staffing (e.g., deployments to/from other jurisdictions), Naval hospital ship Increase support: mental health support	Physicians, nurses	Critical care, emergency medical services, hospitals, primary health care	Observational–descriptive	Challenges (barriers)/enablers (facilitators)
Chaudhary (2017) [43]	Nepal earthquake (2015)	damaged/reduced/insufficient facilities	Increase numbers: solidarity staffing (e.g., deployments to/from other jurisdictions), cross-sector staff deployments Increase flexibility: cross-sector deployments	Physicians, nurses, midwives	Community health services, hospitals, other	Systematic review	Data about patients/procedures; data about workforce; lessons learned
Fredricks (2017) [44]	Nepal earthquake (2015)	Unmet health and social needs	Increase flexibility: new roles, expanded roles	Community health workers	Community health services	Analytical–qualitative	Experiences of workers
Kondo (2019) [45]	Japan earthquake (2016)	Damaged/reduced/insufficient facilities, mass casualty/patient surge, unmet health and social needs	Increase numbers: solidarity staffing (e.g., deployments to/from other jurisdictions), cross-sector staff deployments, Emergency Medical Relief	Physicians, nurses	Critical care, emergency medical services, hospitals, public health	Observational–descriptive	Data about workforce, data about patients/procedures, lessons learned
Lane (2006) [46]	Indian Ocean earthquake/tsunamis (2004)	Damaged/reduced/insufficient facilities, mass casualty/patient surge, unmet health and social needs	Increase numbers: solidarity staffing (e.g., deployments to/from other jurisdictions) Increase flexibility: expanded roles	Physicians, nurses, military health workers, dental workers	Community health services, emergency medical services, other	Observational–descriptive	Data about workforce, lessons learned
Manning (2006) [47]	Indian Ocean earthquake/tsunamis (2004)	Unmet health and social needs	Increase numbers: solidarity staffing (e.g., deployments to/from other jurisdictions) Increase support: mental health services	Community health workers, mental health workers, social workers	Community health services, mental health services, other	Observational–descriptive	Challenges, lessons learned
Roshchin (2002) [48]	India earthquake (2001)	Damaged/reduced/insufficient facilities, mass casualty/patient surge, unmet health and social needs, reduced workforce	Increase numbers: solidarity staffing (e.g., deployments to/from other jurisdictions), international humanitarian emergency response/relief	Physicians, nurses, paramedics	Critical care, emergency medical services, hospitals, primary health care, public health	Observational–descriptive	Data about patients/procedures, challenges (problems), lessons learned
Waxman (2006) [49]	Indian Ocean earthquake/tsunamis (2004)	Damaged/reduced/insufficient facilities, mass casualty/patient surge, unmet health and social needs	Increase numbers: solidarity staffing (e.g., deployments to/from other jurisdictions)	Physicians, nurses, military health workers, public health workers	Emergency medical services, hospitals	Observational–descriptive	Data about patients/procedures, data about workforce, lessons learned

### Types of challenges described

Each of the included articles described one or more disaster-related challenges that could have an impact on the health workforce. The majority of disasters reported mass casualty or patient surge (23/37, 62%), and/or unmet health or social needs (23/37, 62%), and/or damaged, reduced, or otherwise insufficient facilities (17/37, 45%). Other categories of challenges included reduced workforce (6/37, 16%) or staff surplus (due to reduced services) (2/37, 5%). The infrastructural impacts were predominantly seen in extreme weather events or natural disasters and were not often reported in infectious disease outbreaks. Two of the infectious disease papers did report challenges related to reduced facilities: one related to the closure of ICU beds due to personnel shortages during SARS, while the other reported on the Ebola crisis in West Africa and cited challenges related to chronically underdeveloped health infrastructure in that region.

Patient surges were seen across disaster types, though infectious disease events were more likely to give rise to a surge in patients for diagnosis or treatment of that disease, whereas natural disasters and extreme weather events gave rise to a diverse slate of patient complaints related to emergent conditions, such as mass casualty and physical trauma. Unmet health and social needs referred to non-acute care conditions and were also reported across disaster types. These commonly referred to pre-existing conditions which were exacerbated by the disaster itself or through reduced access to health services. This category also comprised the papers reporting on the various non-acute care needs experienced by evacuees and shelter services. Two different workforce challenges were described: staff shortages (usually due to absenteeism) and staff excesses (usually arising due to a reduction or closure of services).

Taken together, the challenges described from the identified literature depict an increase in the demand for health services and a simultaneous decrease in the availability of the workforce (especially relative to the increasing demand).

### Workforce strategies responding to situational challenges

Since the challenges described in the included articles aligned with the challenges we considered in developing our conceptual framework, the response strategies described in these articles were coded deductively into the three capacity levers: *increasing numbers* of workers within a cadre, *increasing the flexibility* of workers across cadres, or *increasing the support* of workers in practice. Most of the papers reported more than one strategy within one or more capacity levers.

The majority of articles reported on strategies used to *increase numbers of health workers* available within the area of need (30/37, 81%). These strategies related to the ability of workers to do their usual occupation or duties but in a different location, where need was high. This type of response was used to address challenges related to infrastructural insufficiency, patient surge, unmet needs, and workforce shortages. A number of strategies fell into this category: solidarity staffing (i.e., deployments to/from other jurisdictions), broadening of scopes of practice to permit workers to staff areas of need, cross-sector staff deployments, the construction and staffing of auxiliary facilities (field hospitals or extensions—*n* = 9, temporary shelters—*n* = 6, mobile medical services—*n* = 2, etc.), increasing work hours, and using volunteers. Nineteen of the papers described solidarity staffing measures, where medical teams or health workers were deployed outside of their home jurisdiction; many of these leveraged existing formal emergency response team structures. Solidarity staffing was most commonly seen for response to weather events and natural disasters, but one paper described the deployment of a Cuban medical team to respond to an Ebola outbreak in West Africa [13]. Bolstering the availability of health services within a more local context leveraged smaller scale staff deployments: for example, cross-sector or inter-departmental deployments of staff within a given organization. The use of auxiliary facilities was seen across disaster types to expand physical capacity and augment the available workforce accordingly; both infrastructural insufficiencies or patient surges were challenges that gave rise to such alternative diagnosis and treatment locations.

Workforce strategies that *increased the flexibility of the workforce* were seen alone or working in concert with aforementioned strategies to increase numbers. These strategies related to the ability of existing workers to do something different from their typical occupation, whether through expanded roles, new roles, rapid up-skilling and/or reskilling, task shifting or delegation, or through cross-sector deployments. This capacity lever also captures the redeployment of staff whose duties are temporarily suspended or who are at-risk due to pre-existing health conditions, as well as virtual care and telehealth solutions. Many of the strategies that increased the flexibility of the health workforce worked synergistically with strategies to increase numbers, resulting in health workers both doing different work and working outside of their usual location.

The final capacity lever captured workforce strategies that *increased support for workers in practice*. Strategies of this type aimed to increase the availability of the existing workforce by supporting them in a variety of ways: through mental health support (including counseling, enforced down time, wellness services, etc.), or by providing housing, transportation assistance, or childcare services. These supportive strategies were meant to reduce staff absenteeism for reasons other than acute illness.

### Types of healthcare providers and settings

**Fig. 5:**
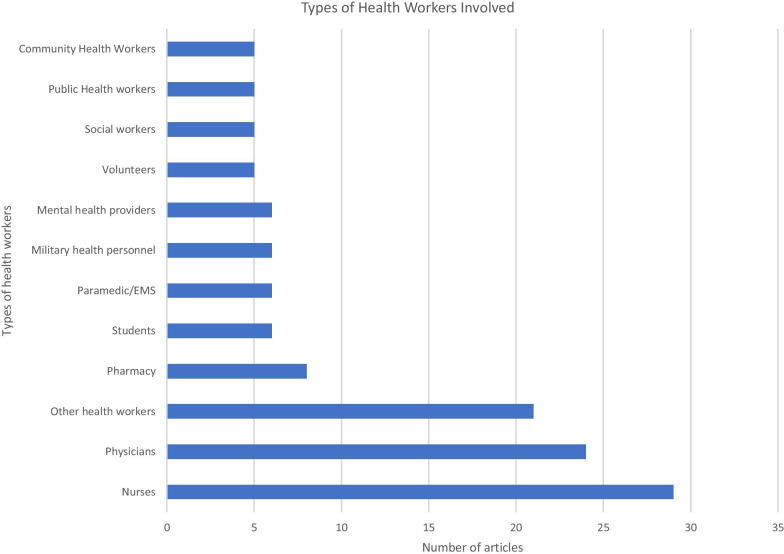
Number of articles mentioning strategies targeting different types of health workers

Most of the included articles discussed workforce strategies related to nurses (30/37, 81%) and/or physicians (26/37, 70%), but a number of other types of health workers are also included (Fig. 5). Organizational settings spanned the gamut from hospital-based settings (critical care, emergency care services), through diagnostic and outpatient care settings, to community and public health settings.

Most of the strategies captured in the included literature described domestic, regional responses to locally situated workforce crises (23/37, 62%); however, a few of the locations described within this group were geographically removed from the actual emergency itself. For example, a few of the strategies in the literature described regional efforts to bolster the workforce serving evacuees at airports and distant shelters. Four of the papers related to domestic mission work (11%), where distant workers were deployed to the site of the emergency.

Nine of the ten international responses described in our literature were related to mission work (9/37, 24%)—where health workers from distant countries were deployed to assist at disaster or emergency sites. One of the papers described a regional international response (3%), where countries in the vicinity of an emergency sent health workers to support their neighbors.

### Barriers and facilitators faced in the implementation of workforce strategies

The literature we evaluated reported a number of barriers and facilitators to the implementation of the workforce strategies that they described. Communication issues related to either infrastructural or organizational factors were reported in 12 of the 37 papers (32%) and were the most frequently reported barriers to efficient emergency workforce response. Eight authors (22%) reported supply issues related to medical equipment, medications, and/or protective equipment. The emotional and physical burden on health workers was described as a barrier by seven authors (19%). Authors who discussed mission-type strategies described barriers related to integration with local services, culture and context (5/37 14%), and a lack of self-sufficiency of deployed teams (3/37, 8%).

A variety of organizational challenges (15/37, 41%) were reported related to the management of patients, scheduling and credentialing of workers, and the coordination of volunteers. Authors noted that a lack of internal structures and processes was a barrier, but that overly restrictive or prescriptive internal structures also impeded response. Internal structures were reported as enablers of successful response by nine authors (24%).

Partnerships and relationships with other response organizations or with local services was frequently cited as a facilitator of emergency response (9/37, 24%). In seven articles (19%), structural factors external to the responding organization were seen to be enablers of workforce strategies; these included factors, such as emergency licensing and credentialing regulations. Formal programs with established organizational processes and structures were seen to be positive factors in seven workforce strategies (19%).

Several workforce strategies described in our included literature identified individual level factors as being important to their ability to respond to the workforce stresses. Many of the strategies relied on medical volunteerism (8/37, 22%) to sustain their response. In such situations, trained and licensed volunteers from regional or distant systems volunteered their services outside of a formal deployment organization, often without training in disaster response. Seven of the articles (19%) identified personal attributes of health workers (e.g., sense of duty, resilience, and flexibility) as being key factors in the successful implementation of workforce strategies.

## Discussion

A number of health workforce strategies emerged from this rapid review of the pre-COVID emergency response literature. The health worker cadres implicated in these strategies reflect the size of the professions and their likelihood of being crisis responders. The strategies found in the literature align well with our COVID-specific conceptual framework, and readily fit into the conceptual categories of workforce capacity levers (increasing numbers, increasing flexibility, and increasing support). In addition to validating the framework for use in our COVID-related workforce innovations project, its suitability for these other types of disasters suggests that the conceptual model may have heuristic value and transferability across multiple health disaster types. We hope future research of this kind can build upon this heuristic framework.

### Lack of evaluative research

Our rapid review highlights how little evidence was available to healthcare managers and decisionmakers in developing workforce strategies to respond to COVID-19. Despite identifying common challenges experienced across multiple types of emergencies affecting the healthcare system, few papers provided analysis of their effectiveness or their impact. Indeed, what was most notable about this literature was the lack of any evaluative content, not just in the crisis phase but also well into the post-crisis phase. That is, a substantial number of the corpus of literature consisted of personal recollections of health care workers’ responses to emergencies presented in narrative format. While these stories, rich in experiential data, lend themselves well to phenomenological or narrative research methods, most did not objectively describe workforce strategies or evaluate outcomes. Although there were few analytical papers reporting outcomes or evaluations, many reflected on impact in some way—either through a quantitative description of the population treated and services provided, or through a reflection on the lessons learned or the factors that helped or hindered the efforts.

In a recently published review of surge capacity workforce strategies related to COVID-19 and other infectious respiratory disease outbreaks, the authors report an emergence of studies which include a more evaluative component [50]. Gupta et al. included evidence generated from simulated experiments and included disaster preparedness evaluations in addition to actual crisis responses [50]. Another notable difference between findings from our pre-COVID study and the emerging COVID research is that our literature included few papers that leveraged virtual care, which, of course, became a key strategy in sustaining access to care during the COVID pandemic [51]. Future evaluative work on the impacts and consequences of this rapid virtual care adoption will be beneficial.

### Limitations

Our rapid scoping review approach had as its primary goal to review the extant literature on emergency and disaster-related workforce strategies and to deploy the knowledge as quickly and widely as possible to support health systems managers and decision-makers responding to the COVID-19 pandemic in Canada. The rapid nature of this request required some methodological compromises which we outlined in our registered protocol [11] and which we acknowledge here. First, our time constraints did not permit review of the extensive corpus of literature in duplicate, which may have resulted in differential application of the inclusion and exclusion criteria despite. We believe the risks of this to be relatively low given that the second phase of screening (full-text) was done in duplicate. Second, we excluded papers for which a full-text copy could not be obtained within the time constraints of the project. This resulted in the exclusion of 100 papers, some of which may have met inclusion criteria and been included in the final analysis.

## Conclusion

Our work exposes a key weakness of the existing literature on workforce responses to disasters and emergencies: the lack of evaluation of impact and effectiveness. We hope that the emerging COVID-related literature will overcome this historical shortcoming and produce evidence of effective workforce strategies, including their impacts on patients, providers, and health systems. Our conceptual framework should provide a solid foundation for identifying and categorizing health work force strategies. Future work may leverage this framework to support a holistic approach towards enhancing health workforce resilience and sustainability.

## Supplementary Information

**Additional file 1.** Final literature search strategies.

## Data Availability

All data generated or analysed during this study are included in this published article and its Additional file 1.

## Abbreviations

CHWN: Canadian Health Workforce Network
ICU: Intensive Care Unit
SARS: Severe Acute Respiratory Syndrome
USA: United States of America

## Footnotes

1 At the time, Healthcare Excellence Canada was working under its previous title of the Canadian Foundation for Healthcare Improvement.
