# Supplementary material for: Health workforce strategies in response to major health events: a rapid scoping review with lessons learned for the response to the COVID-19 pandemic
Source: Hum Resour Health. 2021 Dec 20;19:154. doi: 10.1186/s12960-021-00698-6 (PMC8685817; doi:10.1186/s12960-021-00698-6)
Supplement: Supplementary file 1 — Additional file 1. Final literature search strategies. [file 12960_2021_698_MOESM1_ESM.docx]

**Health workforce strategies in response to major health events: A rapid scoping review with lessons learned for the response to the COVID-19 pandemic**

**Final Literature search strategies (Additional File 1)**

**Literature search methods:**

A peer reviewed search strategy*(McGowan et al.) was conducted April 23, 2020. MEDLINE and Embase were searched via the Ovid platform, and CINAHL was searched via the EBSCO platform. The search was limited to English or French language articles published from 2000 to present. The final search strategy resulted in 3583 records for title and abstract screening after deduplication.

* McGowan J, Sampson M, Salzwedel DM, Cogo E, Foerster V, Lefebvre C. PRESS Peer Review of Electronic Search Strategies: 2015 guideline statement. J Clin Epidemiol. 2016 Jul;75:40-6. <http://www.sciencedirect.com/science/article/pii/S0895435616000585>

**Databases:** Medline, Embase, CINAHL

**Limits:** 2000 to current; English or French language

Results presented in formats for Covidence and Zotero/Endnote - RIS formatted text file exported from Covidence.

**Date run:** April 23, 2020

Medline: 1553

Embase: 1564

CINAHL: 2024

Results after de-duplication: 3583

**Ovid MEDLINE(R) ALL <1946 to April 22, 2020>**

Search history sorted by search number ascending

| **#** | **Searches** | **Results** |
| --- | --- | --- |
|  |  |  |
| 1 | exp coronavirus infections/ | 10865 |
| 2 | exp coronavirus/ | 12453 |
| 3 | (coronavirus* or corona-virus* or covid).ti,ab. | 16954 |
| 4 | or/1-3 | 24531 |
| 5 | limit 4 to yr="2019 -Current" | 7418 |
| 6 | (((wuhan or shanghai or seafood or beijing) and (coronavirus* or corona-virus* or covid)) or 2019-nCoV or 2019nCoV or nCoV2019 or nCov-2019 or COVID-19 or COVID19 or SARS-CoV-2 or HCoV-19).ti,ab. | 6103 |
| 7 | (((coronavirus* or covid or corona-virus* or HCoV or CoV or betacoronavirus*) adj3 (new or novel or "2019" or Wuhan or Hubei or China or Chinese)) or ((Wuhan or Hubei) adj5 pneumonia*)).ti,ab. | 3470 |
| 8 | middle east respiratory syndrome coronavirus/ | 1019 |
| 9 | ((mers adj5 (coronavirus* or virus* or disaster* or crisis or emergency or outbreak*)) or mers-cov or middle east respiratory or middle east virus).ti,ab. | 2328 |
| 10 | sars virus/ | 2981 |
| 11 | ((sars adj5 (coronavirus* or virus* or disaster* or crisis or emergency or outbreak*)) or sars-cov or severe acute respiratory syndrome*).ti,ab. | 7617 |
| 12 | or/5-11 | 15533 |
| 13 | Hemorrhagic Fever, Ebola/ | 5304 |
| 14 | Ebolavirus/ | 3173 |
| 15 | (ebola or ebolavirus).ti,ab. | 8408 |
| 16 | or/13-15 | 9012 |
| 17 | exp disasters/ | 84825 |
| 18 | epidemics/ or pandemics/ or (epidemic* or pandemic*).ti,ab. | 126429 |
| 19 | or/17-18 | 209550 |
| 20 | and/16,19 | 2242 |
| 21 | ((ebola or ebolavirus) adj3 (crisis or emergency or outbreak* or disaster*)).ti,ab. | 1930 |
| 22 | exp natural disasters/ | 16858 |
| 23 | tsunamis/ | 874 |
| 24 | volcanic eruptions/ | 1051 |
| 25 | (avalanche* or cyclonic storm* or cyclone* or drought* or earthquake* or flood or floods or flooding or landslide* or tidal wave* or tsunami* or tornado* or wildfire* or wild-fire* or forest fire* or volcano* or volcanic erupt* or hurricane*).ti,ab. | 55452 |
| 26 | or/22-25 | 59062 |
| 27 | pandemics/ or pandemic*.ti,ab. | 27246 |
| 28 | epidemics/ or epidemic*.ti,ab. | 103268 |
| 29 | exp disasters/ or disaster*.ti,ab. | 95091 |
| 30 | and/28-29 | 1132 |
| 31 | or/12,20-21,26-27,30 | 102552 |
| 32 | Surge Capacity/ | 239 |
| 33 | training support/ | 6077 |
| 34 | (surge capacit* or medical surge*).ti,ab. | 588 |
| 35 | exp Licensure/ | 17495 |
| 36 | licensure*.ti,ab. | 5178 |
| 37 | (redeploy* or re-deploy*).ti,ab. | 630 |
| 38 | ((fasttrack* or fast-track*) adj5 (trainee* or trained or credential* or graduate* or student*)).ti,ab. | 20 |
| 39 | ((Interjurisdictional or jurisdictional or geographic) adj2 mobility).ti,ab. | 166 |
| 40 | (alternat* adj3 deploy*).ti,ab. | 95 |
| 41 | task shift*.ti,ab. | 965 |
| 42 | (skill* adj2 mix).ti,ab. | 1055 |
| 43 | (upskill* or up-skill*).ti,ab. | 270 |
| 44 | (reskill* or re-skill*).ti,ab. | 21 |
| 45 | or/32-44 | 30344 |
| 46 | exp health personnel/ | 507490 |
| 47 | health workforce/ | 12923 |
| 48 | exp administrative personnel/ or social workers/ or exp laboratory personnel/ or exp foreign professional personnel/ or exp emergency responders/ | 61982 |
| 49 | community health workers/ or exp dental auxiliaries/ or home health aides/ or licensed practical nurses/ or exp medical secretaries/ or exp nursing assistants/ or operating room technicians/ or pharmacy technicians/ or physical therapist assistants/ or exp physician assistants/ or caregivers/ or exp dental staff/ or exp dentists/ or doulas/ or school teachers/ or social workers/ or visitors to patients/ or exp volunteers/ | 116634 |
| 50 | Caregivers/ | 35738 |
| 51 | exp education, professional/ or exp education, graduate/ or exp education, medical/ or exp education, nursing/ or exp teaching/ | 335741 |
| 52 | ((community health or home health or personal support or community support or nursing or nurse* or community care or frontline or front-line or emergency) adj2 (worker* or aide* or assistant* or volunteer*)).ti,ab. | 12598 |
| 53 | ((health* or medical or community care or community health or community support or personal support or frontline or front-line or emergency) adj5 (worker* or workforce or professional* or provider* or personnel or volunteer*)).ti. | 58516 |
| 54 | (technician* or technologist* or therapist* or paramedic* or EMT or physician* or doctor* or general practitioner* or nurse practitioner* or specialist* or physiotherapist* or social worker* or support worker* or teacher* or volunteer* or caregiver* or allergist* or anesthesiologist* or anesthetist* or cardiologist* or dental* or dentist* or dermatologist* or endocrinologist* or gastroenterologist* or geriatrician* or hospitalist* or nephrologist* or nurse or nurses or rheumatologist* or neurologist or oncologist* or hematologist* or haematologist* or ophthalmologist* or otolaryngologist* or pathologist* or neonatologist* or pediatrician* or paediatrician* or respirologist* or physiatrist* or pulmonologist* or pharmacist* or psychologist* or psychiatrist* or radiologist* or urologist* or neurosurgeon* or surgeon* or scientist* or lifeguard* or life-guard* or flight attendant*).ti,ab. | 1829627 |
| 55 | ((medical or medicine or nurs* or recent) adj3 (student* or graduate*)).ti,ab. | 78382 |
| 56 | or/46-55 | 2331223 |
| 57 | disaster planning/ | 14036 |
| 58 | health resources/ | 13054 |
| 59 | resource allocation/ | 8300 |
| 60 | capacity building/ | 2455 |
| 61 | exp Professional Role/ | 83368 |
| 62 | Inservice Training/ | 20149 |
| 63 | Delegation, Professional/ | 572 |
| 64 | personnel management/ | 16041 |
| 65 | personnel administration, hospital/ | 5650 |
| 66 | personnel delegation/ | 308 |
| 67 | personnel downsizing/ | 605 |
| 68 | personnel turnover/ | 5140 |
| 69 | exp "personnel staffing and scheduling"/ | 40469 |
| 70 | work engagement/ | 341 |
| 71 | return to work/ | 2435 |
| 72 | Telemedicine/ | 21855 |
| 73 | (mobile health* or telehealth or tele-health or telemedicine or tele-medicine or ehealth or e-health or virtual health or mobile deliver*).ti,ab. | 20802 |
| 74 | (fasttrack* or fast-track*).ti,ab. | 3884 |
| 75 | (task* adj3 (shift* or chang*)).ti,ab. | 6772 |
| 76 | ((new or expanding or shift*) adj2 (role* or job* or task* or skill*)).ti,ab. | 21864 |
| 77 | ((reschedul* or re-schedul* or re-organiz* or reorganiz* or reorganis* or re-organis*) adj2 (shift or shifts)).ti,ab. | 23 |
| 78 | (return-to-work or returning employee* or returning worker* or workers returning).ti,ab. | 9015 |
| 79 | (retiree* or retired or inactive).ti,ab. | 101537 |
| 80 | or/57-79 | 368553 |
| 81 | and/31,45 | 380 |
| 82 | and/31,56,80 | 1372 |
| 83 | or/81-82 | 1717 |
| 84 | limit 83 to (yr="2000 -Current" and (english or french)) | 1553 |

**Embase Classic+Embase <1947 to 2020 April 22>**

Search history sorted by search number ascending

| **#** | **Searches** | **Results** |
| --- | --- | --- |
|  |  |  |
| 1 | exp coronavirinae/ | 13223 |
| 2 | exp coronavirus infection/ | 11968 |
| 3 | (coronavirus* or corona-virus* or covid).ti,ab. | 16325 |
| 4 | or/1-3 | 27156 |
| 5 | limit 4 to yr="2019 -Current" | 6450 |
| 6 | (((wuhan or shanghai or seafood or beijing) and (coronavirus* or corona-virus* or covid)) or 2019-nCoV or 2019nCoV or nCoV2019 or nCov-2019 or COVID-19 or COVID19 or SARS-CoV-2 or HCoV-19).ti,ab. | 4359 |
| 7 | (((coronavirus* or corona-virus* or covid or HCoV or CoV or betacoronavirus*) adj3 (new or novel or "2019" or Wuhan or Hubei or China or Chinese)) or ((Wuhan or Hubei) adj5 pneumonia*)).ti,ab. | 2925 |
| 8 | Middle East respiratory syndrome coronavirus/ | 1963 |
| 9 | ((mers adj5 (coronavirus* or virus* or crisis or emergency or outbreak* or disaster*)) or mers-cov or middle east respiratory or middle east virus).ti,ab. | 2495 |
| 10 | exp sars-related coronavirus/ | 5756 |
| 11 | ((sars adj5 (coronavirus* or virus* or crisis or emergency or outbreak* or disaster*)) or sars-cov or severe acute respiratory syndrome*).ti,ab. | 7645 |
| 12 | or/5-11 | 15874 |
| 13 | Ebola hemorrhagic fever/ | 5676 |
| 14 | exp ebolavirus/ | 3209 |
| 15 | (ebola or ebolavirus).ti,ab. | 9584 |
| 16 | or/13-15 | 11132 |
| 17 | exp disaster/ | 30297 |
| 18 | pandemic/ or pandemic*.ti,ab. | 32294 |
| 19 | epidemic/ or epidemic*.ti,ab. | 188375 |
| 20 | or/17-19 | 242080 |
| 21 | and/16,20 | 4119 |
| 22 | ((ebola or ebolavirus) adj3 (crisis or emergency or outbreak* or disaster*)).ti,ab. | 2166 |
| 23 | natural disaster/ | 2918 |
| 24 | avalanche/ or earthquake/ or tsunami/ or volcano/ or hurricane/ or tornado/ or wildfire/ or forest fire/ or flooding/ | 24785 |
| 25 | (avalanche* or cyclonic storm* or cyclone* or drought* or earthquake* or flood or floods or flooding or landslide* or tidal wave* or tsunami* or tornado* or wildfire* or wild-fire* or forest fire* or volcano* or volcanic erupt* or hurricane*).ti,ab. | 58393 |
| 26 | or/23-25 | 66188 |
| 27 | pandemic/ or pandemic*.ti,ab. | 32294 |
| 28 | epidemic/ or epidemic*.ti,ab. | 188375 |
| 29 | exp disaster/ or disaster*.ti,ab. | 44811 |
| 30 | and/28-29 | 1571 |
| 31 | or/12,21-22,26-27,30 | 117444 |
| 32 | surge capacity/ | 337 |
| 33 | (surge capacit* or medical surge*).ti,ab. | 705 |
| 34 | *licensing/ | 8617 |
| 35 | licensure*.ti,ab. | 5840 |
| 36 | (redeploy* or re-deploy*).ti,ab. | 794 |
| 37 | ((fasttrack* or fast-track*) adj5 (trainee* or trained or credential* or graduate* or student*)).ti,ab. | 22 |
| 38 | ((Interjurisdictional or jurisdictional or geographic) adj2 mobility).ti,ab. | 180 |
| 39 | (alternat* adj3 deploy*).ti,ab. | 118 |
| 40 | task shift*.ti,ab. | 1201 |
| 41 | (skill* adj2 mix).ti,ab. | 1239 |
| 42 | (upskill* or up-skill*).ti,ab. | 570 |
| 43 | (reskill* or re-skill*).ti,ab. | 21 |
| 44 | or/32-43 | 17892 |
| 45 | exp health care personnel/ | 1594923 |
| 46 | flight attendant/ | 48 |
| 47 | school teacher/ | 1302 |
| 48 | social worker/ | 12747 |
| 49 | fire fighter/ | 3031 |
| 50 | police/ | 12926 |
| 51 | caregiver/ | 79056 |
| 52 | layperson/ | 518 |
| 53 | exp volunteer/ | 64019 |
| 54 | exp student/ | 262896 |
| 55 | laboratory personnel/ | 5962 |
| 56 | ((community health or home health or personal support or community support or nursing or nurse* or community care or frontline or front-line or emergency) adj2 (worker* or aide* or assistant* or volunteer*)).ti,ab. | 16399 |
| 57 | ((health* or medical or community care or community health or community support or personal support or frontline or front-line or emergency) adj5 (worker* or workforce or professional* or provider* or personnel or volunteer*)).ti. | 71888 |
| 58 | (technician* or technologist* or therapist* or paramedic* or EMT or physician* or doctor* or general practitioner* or nurse practitioner* or specialist* or physiotherapist* or social worker* or support worker* or teacher* or volunteer* or caregiver* or allergist* or anesthesiologist* or anesthetist* or cardiologist* or dental* or dentist* or dermatologist* or endocrinologist* or gastroenterologist* or geriatrician* or hospitalist* or nephrologist* or nurse or nurses or rheumatologist* or neurologist or oncologist* or hematologist* or haematologist* or ophthalmologist* or otolaryngologist* or pathologist* or neonatologist* or pediatrician* or paediatrician* or respirologist* or physiatrist* or pulmonologist* or pharmacist* or psychologist* or psychiatrist* or radiologist* or urologist* or neurosurgeon* or surgeon* or scientist* or lifeguard* or life-guard* or flight attendant*).ti,ab. | 2577775 |
| 59 | ((medical or medicine or nurs* or recent) adj3 (student* or graduate*)).ti,ab. | 96656 |
| 60 | or/45-59 | 3439830 |
| 61 | disaster planning/ | 12382 |
| 62 | resource allocation/ | 20379 |
| 63 | capacity building/ | 4793 |
| 64 | "scope of practice"/ | 1994 |
| 65 | in service training/ | 16262 |
| 66 | professional delegation/ | 1331 |
| 67 | hospital personnel management/ | 580 |
| 68 | health care personnel management/ | 1628 |
| 69 | *personnel management/ | 32527 |
| 70 | work engagement/ | 1002 |
| 71 | return to work/ | 6011 |
| 72 | exp *telehealth/ | 23538 |
| 73 | (mobile health* or telehealth or tele-health or telemedicine or tele-medicine or ehealth or e-health or virtual health or mobile deliver*).ti,ab. | 26637 |
| 74 | (fasttrack* or fast-track*).ti,ab. | 6160 |
| 75 | (task* adj3 (shift* or chang*)).ti,ab. | 8160 |
| 76 | ((new or expanding or shift*) adj2 (role* or job* or task* or skill*)).ti,ab. | 27673 |
| 77 | ((reschedul* or re-schedul* or re-organiz* or reorganiz* or reorganis* or re-organis*) adj2 (shift or shifts)).ti,ab. | 24 |
| 78 | (return-to-work or returning employee* or returning worker* or workers returning).ti,ab. | 12123 |
| 79 | (retiree* or retired or inactive).ti,ab. | 130850 |
| 80 | or/61-79 | 311356 |
| 81 | and/31,44 | 397 |
| 82 | and/31,60,80 | 1333 |
| 83 | or/81-82 | 1685 |
| 84 | limit 83 to ((english or french) and yr="2000 -Current") | 1564 |

Ovid multi-database search (as run) – Medline and Embase:

| **Embase, Ovid MEDLINE(R)**  Search history sorted by search number ascending   \| **#** \| **Searches** \| **Results** \| \| --- \| --- \| --- \| \|  \|  \|  \| \| 1 \| exp coronavirus infections/ \| 22833 \| \| 2 \| exp coronavirus/ \| 25676 \| \| 3 \| (coronavirus* or corona-virus* or covid).ti,ab. \| 33279 \| \| 4 \| or/1-3 \| 51687 \| \| 5 \| limit 4 to yr="2019 -Current" \| 13868 \| \| 6 \| (((wuhan or shanghai or seafood or beijing) and (coronavirus* or corona-virus* or covid)) or 2019-nCoV or 2019nCoV or nCoV2019 or nCov-2019 or COVID-19 or COVID19 or SARS-CoV-2 or HCoV-19).ti,ab. \| 10462 \| \| 7 \| (((coronavirus* or covid or corona-virus* or HCoV or CoV or betacoronavirus*) adj3 (new or novel or "2019" or Wuhan or Hubei or China or Chinese)) or ((Wuhan or Hubei) adj5 pneumonia*)).ti,ab. \| 6395 \| \| 8 \| middle east respiratory syndrome coronavirus/ \| 2982 \| \| 9 \| ((mers adj5 (coronavirus* or virus* or disaster* or crisis or emergency or outbreak*)) or mers-cov or middle east respiratory or middle east virus).ti,ab. \| 4823 \| \| 10 \| sars virus/ \| 8604 \| \| 11 \| ((sars adj5 (coronavirus* or virus* or disaster* or crisis or emergency or outbreak*)) or sars-cov or severe acute respiratory syndrome*).ti,ab. \| 15262 \| \| 12 \| or/5-11 \| 31405 \| \| 13 \| Hemorrhagic Fever, Ebola/ \| 8089 \| \| 14 \| Ebolavirus/ \| 6193 \| \| 15 \| (ebola or ebolavirus).ti,ab. \| 17992 \| \| 16 \| or/13-15 \| 19594 \| \| 17 \| exp disasters/ \| 115122 \| \| 18 \| epidemics/ or pandemics/ or (epidemic* or pandemic*).ti,ab. \| 326997 \| \| 19 \| or/17-18 \| 439356 \| \| 20 \| and/16,19 \| 5145 \| \| 21 \| ((ebola or ebolavirus) adj3 (crisis or emergency or outbreak* or disaster*)).ti,ab. \| 4096 \| \| 22 \| exp natural disasters/ \| 19776 \| \| 23 \| tsunamis/ \| 3184 \| \| 24 \| volcanic eruptions/ \| 4545 \| \| 25 \| (avalanche* or cyclonic storm* or cyclone* or drought* or earthquake* or flood or floods or flooding or landslide* or tidal wave* or tsunami* or tornado* or wildfire* or wild-fire* or forest fire* or volcano* or volcanic erupt* or hurricane*).ti,ab. \| 113845 \| \| 26 \| or/22-25 \| 121390 \| \| 27 \| pandemics/ or pandemic*.ti,ab. \| 58872 \| \| 28 \| epidemics/ or epidemic*.ti,ab. \| 279464 \| \| 29 \| exp disasters/ or disaster*.ti,ab. \| 139902 \| \| 30 \| and/28-29 \| 2531 \| \| 31 \| or/12,20-21,26-27,30 \| 214790 \| \| 32 \| Surge Capacity/ \| 576 \| \| 33 \| training support/ \| 422215 \| \| 34 \| (surge capacit* or medical surge*).ti,ab. \| 1293 \| \| 35 \| exp Licensure/ \| 109678 \| \| 36 \| licensure*.ti,ab. \| 11018 \| \| 37 \| (redeploy* or re-deploy*).ti,ab. \| 1424 \| \| 38 \| ((fasttrack* or fast-track*) adj5 (trainee* or trained or credential* or graduate* or student*)).ti,ab. \| 42 \| \| 39 \| ((Interjurisdictional or jurisdictional or geographic) adj2 mobility).ti,ab. \| 346 \| \| 40 \| (alternat* adj3 deploy*).ti,ab. \| 213 \| \| 41 \| task shift*.ti,ab. \| 2166 \| \| 42 \| (skill* adj2 mix).ti,ab. \| 2294 \| \| 43 \| (upskill* or up-skill*).ti,ab. \| 840 \| \| 44 \| (reskill* or re-skill*).ti,ab. \| 42 \| \| 45 \| or/32-44 \| 532387 \| \| 46 \| exp health personnel/ \| 2102413 \| \| 47 \| health workforce/ \| 13650 \| \| 48 \| exp administrative personnel/ or social workers/ or exp laboratory personnel/ or exp foreign professional personnel/ or exp emergency responders/ \| 122828 \| \| 49 \| community health workers/ or exp dental auxiliaries/ or home health aides/ or licensed practical nurses/ or exp medical secretaries/ or exp nursing assistants/ or operating room technicians/ or pharmacy technicians/ or physical therapist assistants/ or exp physician assistants/ or caregivers/ or exp dental staff/ or exp dentists/ or doulas/ or school teachers/ or social workers/ or visitors to patients/ or exp volunteers/ \| 466747 \| \| 50 \| Caregivers/ \| 92579 \| \| 51 \| exp education, professional/ or exp education, graduate/ or exp education, medical/ or exp education, nursing/ or exp teaching/ \| 854605 \| \| 52 \| ((community health or home health or personal support or community support or nursing or nurse* or community care or frontline or front-line or emergency) adj2 (worker* or aide* or assistant* or volunteer*)).ti,ab. \| 28997 \| \| 53 \| ((health* or medical or community care or community health or community support or personal support or frontline or front-line or emergency) adj5 (worker* or workforce or professional* or provider* or personnel or volunteer*)).ti. \| 130404 \| \| 54 \| (technician* or technologist* or therapist* or paramedic* or EMT or physician* or doctor* or general practitioner* or nurse practitioner* or specialist* or physiotherapist* or social worker* or support worker* or teacher* or volunteer* or caregiver* or allergist* or anesthesiologist* or anesthetist* or cardiologist* or dental* or dentist* or dermatologist* or endocrinologist* or gastroenterologist* or geriatrician* or hospitalist* or nephrologist* or nurse or nurses or rheumatologist* or neurologist or oncologist* or hematologist* or haematologist* or ophthalmologist* or otolaryngologist* or pathologist* or neonatologist* or pediatrician* or paediatrician* or respirologist* or physiatrist* or pulmonologist* or pharmacist* or psychologist* or psychiatrist* or radiologist* or urologist* or neurosurgeon* or surgeon* or scientist* or lifeguard* or life-guard* or flight attendant*).ti,ab. \| 4407402 \| \| 55 \| ((medical or medicine or nurs* or recent) adj3 (student* or graduate*)).ti,ab. \| 175038 \| \| 56 \| or/46-55 \| 5890380 \| \| 57 \| disaster planning/ \| 26418 \| \| 58 \| health resources/ \| 106005 \| \| 59 \| resource allocation/ \| 28679 \| \| 60 \| capacity building/ \| 7248 \| \| 61 \| exp Professional Role/ \| 521246 \| \| 62 \| Inservice Training/ \| 35812 \| \| 63 \| Delegation, Professional/ \| 1859 \| \| 64 \| personnel management/ \| 73951 \| \| 65 \| personnel administration, hospital/ \| 17678 \| \| 66 \| personnel delegation/ \| 58218 \| \| 67 \| personnel downsizing/ \| 58515 \| \| 68 \| personnel turnover/ \| 59522 \| \| 69 \| exp "personnel staffing and scheduling"/ \| 132116 \| \| 70 \| work engagement/ \| 1343 \| \| 71 \| return to work/ \| 8446 \| \| 72 \| Telemedicine/ \| 44844 \| \| 73 \| (mobile health* or telehealth or tele-health or telemedicine or tele-medicine or ehealth or e-health or virtual health or mobile deliver*).ti,ab. \| 47439 \| \| 74 \| (fasttrack* or fast-track*).ti,ab. \| 10044 \| \| 75 \| (task* adj3 (shift* or chang*)).ti,ab. \| 14932 \| \| 76 \| ((new or expanding or shift*) adj2 (role* or job* or task* or skill*)).ti,ab. \| 49537 \| \| 77 \| ((reschedul* or re-schedul* or re-organiz* or reorganiz* or reorganis* or re-organis*) adj2 (shift or shifts)).ti,ab. \| 47 \| \| 78 \| (return-to-work or returning employee* or returning worker* or workers returning).ti,ab. \| 21138 \| \| 79 \| (retiree* or retired or inactive).ti,ab. \| 232387 \| \| 80 \| or/57-79 \| 1237050 \| \| 81 \| and/31,45 \| 1975 \| \| 82 \| and/31,56,80 \| 3771 \| \| 83 \| or/81-82 \| 5437 \| \| 84 \| 83 use medall \| 1717 \| \| 85 \| exp coronavirinae/ \| 26850 \| \| 86 \| exp coronavirus infection/ \| 22833 \| \| 87 \| (coronavirus* or corona-virus* or covid).ti,ab. \| 33279 \| \| 88 \| or/85-87 \| 52021 \| \| 89 \| limit 88 to yr="2019 -Current" \| 13870 \| \| 90 \| (((wuhan or shanghai or seafood or beijing) and (coronavirus* or corona-virus* or covid)) or 2019-nCoV or 2019nCoV or nCoV2019 or nCov-2019 or COVID-19 or COVID19 or SARS-CoV-2 or HCoV-19).ti,ab. \| 10462 \| \| 91 \| (((coronavirus* or corona-virus* or covid or HCoV or CoV or betacoronavirus*) adj3 (new or novel or "2019" or Wuhan or Hubei or China or Chinese)) or ((Wuhan or Hubei) adj5 pneumonia*)).ti,ab. \| 6395 \| \| 92 \| Middle East respiratory syndrome coronavirus/ \| 2982 \| \| 93 \| ((mers adj5 (coronavirus* or virus* or crisis or emergency or outbreak* or disaster*)) or mers-cov or middle east respiratory or middle east virus).ti,ab. \| 4823 \| \| 94 \| exp sars-related coronavirus/ \| 8737 \| \| 95 \| ((sars adj5 (coronavirus* or virus* or crisis or emergency or outbreak* or disaster*)) or sars-cov or severe acute respiratory syndrome*).ti,ab. \| 15262 \| \| 96 \| or/89-95 \| 31409 \| \| 97 \| Ebola hemorrhagic fever/ \| 10980 \| \| 98 \| exp ebolavirus/ \| 6382 \| \| 99 \| (ebola or ebolavirus).ti,ab. \| 17992 \| \| 100 \| or/97-99 \| 20144 \| \| 101 \| exp disaster/ \| 30297 \| \| 102 \| pandemic/ or pandemic*.ti,ab. \| 59540 \| \| 103 \| epidemic/ or epidemic*.ti,ab. \| 291643 \| \| 104 \| or/101-103 \| 368509 \| \| 105 \| and/100,104 \| 6188 \| \| 106 \| ((ebola or ebolavirus) adj3 (crisis or emergency or outbreak* or disaster*)).ti,ab. \| 4096 \| \| 107 \| natural disaster/ \| 3101 \| \| 108 \| avalanche/ or earthquake/ or tsunami/ or volcano/ or hurricane/ or tornado/ or wildfire/ or forest fire/ or flooding/ \| 31713 \| \| 109 \| (avalanche* or cyclonic storm* or cyclone* or drought* or earthquake* or flood or floods or flooding or landslide* or tidal wave* or tsunami* or tornado* or wildfire* or wild-fire* or forest fire* or volcano* or volcanic erupt* or hurricane*).ti,ab. \| 113845 \| \| 110 \| or/107-109 \| 123199 \| \| 111 \| pandemic/ or pandemic*.ti,ab. \| 59540 \| \| 112 \| epidemic/ or epidemic*.ti,ab. \| 291643 \| \| 113 \| exp disaster/ or disaster*.ti,ab. \| 69218 \| \| 114 \| and/112-113 \| 2116 \| \| 115 \| or/96,105-106,110-111,114 \| 217568 \| \| 116 \| surge capacity/ \| 576 \| \| 117 \| (surge capacit* or medical surge*).ti,ab. \| 1293 \| \| 118 \| *licensing/ \| 12198 \| \| 119 \| licensure*.ti,ab. \| 11018 \| \| 120 \| (redeploy* or re-deploy*).ti,ab. \| 1424 \| \| 121 \| ((fasttrack* or fast-track*) adj5 (trainee* or trained or credential* or graduate* or student*)).ti,ab. \| 42 \| \| 122 \| ((Interjurisdictional or jurisdictional or geographic) adj2 mobility).ti,ab. \| 346 \| \| 123 \| (alternat* adj3 deploy*).ti,ab. \| 213 \| \| 124 \| task shift*.ti,ab. \| 2166 \| \| 125 \| (skill* adj2 mix).ti,ab. \| 2294 \| \| 126 \| (upskill* or up-skill*).ti,ab. \| 840 \| \| 127 \| (reskill* or re-skill*).ti,ab. \| 42 \| \| 128 \| or/116-127 \| 29964 \| \| 129 \| exp health care personnel/ \| 1594923 \| \| 130 \| flight attendant/ \| 48 \| \| 131 \| school teacher/ \| 2621 \| \| 132 \| social worker/ \| 13318 \| \| 133 \| fire fighter/ \| 4053 \| \| 134 \| police/ \| 18029 \| \| 135 \| caregiver/ \| 114794 \| \| 136 \| layperson/ \| 518 \| \| 137 \| exp volunteer/ \| 92287 \| \| 138 \| exp student/ \| 389325 \| \| 139 \| laboratory personnel/ \| 6503 \| \| 140 \| ((community health or home health or personal support or community support or nursing or nurse* or community care or frontline or front-line or emergency) adj2 (worker* or aide* or assistant* or volunteer*)).ti,ab. \| 28997 \| \| 141 \| ((health* or medical or community care or community health or community support or personal support or frontline or front-line or emergency) adj5 (worker* or workforce or professional* or provider* or personnel or volunteer*)).ti. \| 130404 \| \| 142 \| (technician* or technologist* or therapist* or paramedic* or EMT or physician* or doctor* or general practitioner* or nurse practitioner* or specialist* or physiotherapist* or social worker* or support worker* or teacher* or volunteer* or caregiver* or allergist* or anesthesiologist* or anesthetist* or cardiologist* or dental* or dentist* or dermatologist* or endocrinologist* or gastroenterologist* or geriatrician* or hospitalist* or nephrologist* or nurse or nurses or rheumatologist* or neurologist or oncologist* or hematologist* or haematologist* or ophthalmologist* or otolaryngologist* or pathologist* or neonatologist* or pediatrician* or paediatrician* or respirologist* or physiatrist* or pulmonologist* or pharmacist* or psychologist* or psychiatrist* or radiologist* or urologist* or neurosurgeon* or surgeon* or scientist* or lifeguard* or life-guard* or flight attendant*).ti,ab. \| 4407402 \| \| 143 \| ((medical or medicine or nurs* or recent) adj3 (student* or graduate*)).ti,ab. \| 175038 \| \| 144 \| or/129-143 \| 5450529 \| \| 145 \| disaster planning/ \| 26418 \| \| 146 \| resource allocation/ \| 28679 \| \| 147 \| capacity building/ \| 7248 \| \| 148 \| "scope of practice"/ \| 2017 \| \| 149 \| in service training/ \| 16262 \| \| 150 \| professional delegation/ \| 1903 \| \| 151 \| hospital personnel management/ \| 6230 \| \| 152 \| health care personnel management/ \| 1628 \| \| 153 \| *personnel management/ \| 44858 \| \| 154 \| work engagement/ \| 1343 \| \| 155 \| return to work/ \| 8446 \| \| 156 \| exp *telehealth/ \| 46794 \| \| 157 \| (mobile health* or telehealth or tele-health or telemedicine or tele-medicine or ehealth or e-health or virtual health or mobile deliver*).ti,ab. \| 47439 \| \| 158 \| (fasttrack* or fast-track*).ti,ab. \| 10044 \| \| 159 \| (task* adj3 (shift* or chang*)).ti,ab. \| 14932 \| \| 160 \| ((new or expanding or shift*) adj2 (role* or job* or task* or skill*)).ti,ab. \| 49537 \| \| 161 \| ((reschedul* or re-schedul* or re-organiz* or reorganiz* or reorganis* or re-organis*) adj2 (shift or shifts)).ti,ab. \| 47 \| \| 162 \| (return-to-work or returning employee* or returning worker* or workers returning).ti,ab. \| 21138 \| \| 163 \| (retiree* or retired or inactive).ti,ab. \| 232387 \| \| 164 \| or/145-163 \| 528418 \| \| 165 \| and/115,128 \| 748 \| \| 166 \| and/115,144,164 \| 2115 \| \| 167 \| or/165-166 \| 2796 \| \| 168 \| 167 use emczd \| 1685 \| \| 169 \| or/84,168 \| 3402 \| \| 170 \| limit 169 to (english or french) \| 3264 \| \| 171 \| limit 170 to yr="2000 -Current" \| 3117 \| \| 172 \| remove duplicates from 171 \| 2212 \| |
| --- | --- | --- | --- | --- | --- | --- | --- | --- | --- | --- | --- | --- | --- | --- | --- | --- | --- | --- | --- | --- | --- | --- | --- | --- | --- | --- | --- | --- | --- | --- | --- | --- | --- | --- | --- | --- | --- | --- | --- | --- | --- | --- | --- | --- | --- | --- | --- | --- | --- | --- | --- | --- | --- | --- | --- | --- | --- | --- | --- | --- | --- | --- | --- | --- | --- | --- | --- | --- | --- | --- | --- | --- | --- | --- | --- | --- | --- | --- | --- | --- | --- | --- | --- | --- | --- | --- | --- | --- | --- | --- | --- | --- | --- | --- | --- | --- | --- | --- | --- | --- | --- | --- | --- | --- | --- | --- | --- | --- | --- | --- | --- | --- | --- | --- | --- | --- | --- | --- | --- | --- | --- | --- | --- | --- | --- | --- | --- | --- | --- | --- | --- | --- | --- | --- | --- | --- | --- | --- | --- | --- | --- | --- | --- | --- | --- | --- | --- | --- | --- | --- | --- | --- | --- | --- | --- | --- | --- | --- | --- | --- | --- | --- | --- | --- | --- | --- | --- | --- | --- | --- | --- | --- | --- | --- | --- | --- | --- | --- | --- | --- | --- | --- | --- | --- | --- | --- | --- | --- | --- | --- | --- | --- | --- | --- | --- | --- | --- | --- | --- | --- | --- | --- | --- | --- | --- | --- | --- | --- | --- | --- | --- | --- | --- | --- | --- | --- | --- | --- | --- | --- | --- | --- | --- | --- | --- | --- | --- | --- | --- | --- | --- | --- | --- | --- | --- | --- | --- | --- | --- | --- | --- | --- | --- | --- | --- | --- | --- | --- | --- | --- | --- | --- | --- | --- | --- | --- | --- | --- | --- | --- | --- | --- | --- | --- | --- | --- | --- | --- | --- | --- | --- | --- | --- | --- | --- | --- | --- | --- | --- | --- | --- | --- | --- | --- | --- | --- | --- | --- | --- | --- | --- | --- | --- | --- | --- | --- | --- | --- | --- | --- | --- | --- | --- | --- | --- | --- | --- | --- | --- | --- | --- | --- | --- | --- | --- | --- | --- | --- | --- | --- | --- | --- | --- | --- | --- | --- | --- | --- | --- | --- | --- | --- | --- | --- | --- | --- | --- | --- | --- | --- | --- | --- | --- | --- | --- | --- | --- | --- | --- | --- | --- | --- | --- | --- | --- | --- | --- | --- | --- | --- | --- | --- | --- | --- | --- | --- | --- | --- | --- | --- | --- | --- | --- | --- | --- | --- | --- | --- | --- | --- | --- | --- | --- | --- | --- | --- | --- | --- | --- | --- | --- | --- | --- | --- | --- | --- | --- | --- | --- | --- | --- | --- | --- | --- | --- | --- | --- | --- | --- | --- | --- | --- | --- | --- | --- | --- | --- | --- | --- | --- | --- | --- | --- | --- | --- | --- | --- | --- | --- | --- | --- | --- | --- | --- | --- | --- | --- | --- | --- | --- | --- | --- | --- | --- | --- | --- | --- | --- | --- | --- | --- | --- | --- | --- | --- | --- | --- | --- | --- | --- | --- | --- | --- | --- | --- | --- | --- | --- | --- | --- | --- | --- | --- | --- | --- | --- | --- | --- | --- | --- | --- | --- | --- | --- | --- | --- | --- | --- | --- | --- | --- | --- | --- | --- | --- | --- | --- | --- | --- | --- | --- | --- | --- | --- | --- | --- | --- | --- | --- | --- | --- | --- | --- | --- | --- | --- | --- | --- | --- | --- | --- | --- |

CINAHL search:

| Thursday, April 23, 2020 4:20:04 PM | | | |  |
| --- | --- | --- | --- | --- |
| **#** | **Query** | **Limiters/Expanders** | **Results** | |
| S22 | S19 OR S20 | Limiters - Published Date: 20000101-20211231; Language: English, French Search modes - Boolean/Phrase | 2,024 | |
| S21 | S19 OR S20 | Search modes - Boolean/Phrase | 2,088 | |
| S20 | S15 AND S17 AND S18 | Search modes - Boolean/Phrase | 2,017 | |
| S19 | S15 AND S16 | Search modes - Boolean/Phrase | 95 | |
| S18 | ( (MH "Disaster Planning+") OR (MH "Resource Allocation") OR (MH "Health Resource Allocation") OR (MH "Health Resource Utilization") OR (MH "Professional Role+") OR (MH "Personnel Management") OR (MH "Delegation of Authority") OR (MH "Personnel Staffing and Scheduling+") OR (MH "Work Engagement") OR (MH "Job Re-Entry") OR (MH "Telehealth+") OR (MH "Personnel Shortage+") ) OR ( TI ( "mobile health*" OR telehealth OR tele-health OR telemedicine OR tele-medicine OR ehealth OR e-health OR "virtual health" OR "mobile deliver*" ) OR TI ( fasttrack* OR fast-track* ) OR TI ( task* N3 (shift* OR chang*) ) OR TI ( (new OR expanding OR shift*) N2 (role* OR job* OR task* OR skill*) ) OR TI ( (reschedul* OR re-schedul* OR re-organiz* OR reorganiz* OR reorganis* OR re-organis*) N2 (shift OR shifts) ) OR TI ( return-to-work OR "returning employee*" OR "returning worker*" Or "workers returning" ) OR TI ( retiree* OR retired OR inactive ) ) OR ( AB ( "mobile health*" OR telehealth OR tele-health OR telemedicine OR tele-medicine OR ehealth OR e-health OR "virtual health" OR "mobile deliver*" ) OR AB ( fasttrack* OR fast-track* ) OR AB ( task* N3 (shift* OR chang*) ) OR AB ( (new OR expanding OR shift*) N2 (role* OR job* OR task* OR skill*) ) OR AB ( (reschedul* OR re-schedul* OR re-organiz* OR reorganiz* OR reorganis* OR re-organis*) N2 (shift OR shifts) ) OR AB ( return-to-work OR "returning employee*" OR "returning worker*" Or "workers returning" ) OR AB ( retiree* OR retired OR inactive ) ) | Search modes - Boolean/Phrase | 272,385 | |
| S17 | ( ( (MH "Health Personnel+") OR (MH "Caregivers") OR (MH "College Graduates") OR (MH "New Graduates+") OR (MH "Clerical Personnel") OR (MH "Correctional Facilities Personnel") OR (MH "Firefighters") OR (MH "Foreign Professional Personnel+") OR (MH "Police") OR (MH "Teachers") OR (MH "Volunteer Workers") ) ) OR ( TI( ("community health" OR "home health" OR "personal support" OR "community support" OR nursing OR nurse* OR "community care" OR frontline OR front-line OR emergency) N2 (worker* OR aide* OR assistant* OR volunteer*) ) OR TI ( (health* OR medical OR "community care" OR "community health" OR "community support" OR "personal support" OR frontline OR front-line OR emergency) N5 (worker* OR workforce OR professional* OR provider* OR personnel OR volunteer*) ) OR TI ( technician* OR technologist* OR therapist* OR paramedic* OR EMT OR physician* OR doctor* OR "general practitioner*" OR "nurse practitioner*" OR specialist* OR physiotherapist* OR "social worker*" OR "support worker*" OR teacher* OR volunteer* OR caregiver* OR allergist* OR anesthesiologist* OR anesthetist* OR cardiologist* OR dental* OR dentist* OR dermatologist* OR endocrinologist* OR gastroenterologist* OR geriatrician* OR hospitalist* OR nephrologist* OR nurse OR nurses OR rheumatologist* OR neurologist OR oncologist* OR hematologist* OR haematologist* OR ophthalmologist* OR otolaryngologist* OR pathologist* OR neonatologist* OR pediatrician* OR paediatrician* OR respirologist* OR physiatrist* OR pulmonologist* OR pharmacist* OR psychologist* OR psychiatrist* OR radiologist* OR urologist* OR neurosurgeon* OR surgeon* OR scientist* OR lifeguard* OR life-guard* OR "flight attendant*" ) OR TI ( ((medical OR medicine OR nurs* OR recent) N3 (student* OR graduate*) ) ) OR ( AB ( ("community health" OR "home health" OR "personal support" OR "community support" OR nursing OR nurse* OR "community care" OR frontline OR front-line OR emergency) N2 (worker* OR aide* OR assistant* OR volunteer*) ) OR AB ( technician* OR technologist* OR therapist* OR paramedic* OR EMT OR physician* OR doctor* OR "general practitioner*" OR "nurse practitioner*" OR specialist* OR physiotherapist* OR "social worker*" OR "support worker*" OR teacher* OR volunteer* OR caregiver* OR allergist* OR anesthesiologist* OR anesthetist* OR cardiologist* OR dental* OR dentist* OR dermatologist* OR endocrinologist* OR gastroenterologist* OR geriatrician* OR hospitalist* OR nephrologist* OR nurse OR nurses OR rheumatologist* OR neurologist OR oncologist* OR hematologist* OR haematologist* OR ophthalmologist* OR otolaryngologist* OR pathologist* OR neonatologist* OR pediatrician* OR paediatrician* OR respirologist* OR physiatrist* OR pulmonologist* OR pharmacist* OR psychologist* OR psychiatrist* OR radiologist* OR urologist* OR neurosurgeon* OR surgeon* OR scientist* OR lifeguard* OR life-guard* OR "flight attendant*" ) OR AB ( ((medical OR medicine OR nurs* OR recent) N3 (student* OR graduate*) ) ) | Search modes - Boolean/Phrase | 1,362,929 | |
| S16 | ( ( (MH "Licensure+") OR TI licensure* ) OR TI ( "surge capacit*" OR "medical surge*" ) OR TI ( redeploy* OR re-deploy* ) OR TI ( (fasttrack* OR fast-track*) N5 (trainee* OR trained OR credential* OR graduate* OR student*) ) OR TI ( (interjurisdictional OR jurisdictional OR geographic) N2 mobility ) OR TI alternat* N3 deploy* OR TI "task shift*" OR TI ( (skill* N2 mix) OR upskill* OR up-skill* OR reskill* OR re-skill* ) ) OR ( AB ( (fasttrack* OR fast-track*) N5 (trainee* OR trained OR credential* OR graduate* OR student*) ) OR AB ( (interjurisdictional OR jurisdictional OR geographic) N2 mobility ) OR AB alternat* N3 deploy* OR TI "task shift*" OR AB ( (skill* N2 mix) OR upskill* OR up-skill* OR reskill* OR re-skill* ) ) | Search modes - Boolean/Phrase | 17,184 | |
| S15 | S1 OR S2 OR S3 OR S4 OR S7 OR S8 OR S9 OR S10 OR S11 OR S14 | Search modes - Boolean/Phrase | 35,068 | |
| S14 | S12 AND S13 | Search modes - Boolean/Phrase | 207 | |
| S13 | TI epidemic* OR AB epidemic* | Search modes - Boolean/Phrase | 23,932 | |
| S12 | (MH "Disasters") OR TI disaster* OR AB disaster* | Search modes - Boolean/Phrase | 17,734 | |
| S11 | TI pandemic* OR AB pandemic* | Search modes - Boolean/Phrase | 7,806 | |
| S10 | ( (MH "Natural Disasters") OR (MH "Fires+") ) OR TI ( avalanche* OR "cyclonic storm*" OR cyclone* OR drought* OR earthquake* OR flood OR floods OR flooding OR landslide* OR "tidal wave*" OR tsunami* OR tornado* OR wildfire* OR wild-fire* OR "forest fire*" OR volcano* OR "volcanic erupt*" OR hurricane* ) OR AB ( avalanche* OR "cyclonic storm*" OR cyclone* OR drought* OR earthquake* OR flood OR floods OR flooding OR landslide* OR "tidal wave*" OR tsunami* OR tornado* OR wildfire* OR wild-fire* OR "forest fire*" OR volcano* OR "volcanic erupt*" OR hurricane* ) | Search modes - Boolean/Phrase | 21,744 | |
| S9 | TI ( (SARS OR MERS) N3 (disaster* OR crisis OR outbreak* or emergency) ) OR AB ( (SARS OR MERS) N3 (disaster* OR crisis OR outbreak* or emergency) ) | Search modes - Boolean/Phrase | 462 | |
| S8 | TI ( (ebola OR ebolavirus) N3 (disaster* OR crisis OR outbreak* or emergency) ) OR AB ( (ebola OR ebolavirus) N3 (disaster* OR crisis OR outbreak* or emergency) ) | Search modes - Boolean/Phrase | 1,103 | |
| S7 | S5 AND S6 | Search modes - Boolean/Phrase | 828 | |
| S6 | ( (MH "Disasters") OR (MH "Mass Casualty Incidents") ) OR ( TI ( pandemic* OR epidemic* ) ) OR ( AB ( pandemic* OR epidemic* ) ) | Search modes - Boolean/Phrase | 39,897 | |
| S5 | ( (MH "Hemorrhagic Fever, Ebola") OR (MH "Ebola Virus") ) OR ( TI ( ebola OR ebolavirus ) ) OR ( AB ( ebola OR ebolavirus ) ) | Search modes - Boolean/Phrase | 5,316 | |
| S4 | ( ( (MH "SARS Virus") OR (MH "Severe Acute Respiratory Syndrome") ) ) OR ( TI ( (sars N5 (coronavirus* OR corona-virus* OR virus*)) OR sars-cov OR "severe acute respiratory syndrome*" ) ) OR ( AB ( (sars N5 (coronavirus* OR corona-virus* OR virus*)) OR sars-cov OR "severe acute respiratory syndrome*" ) ) | Search modes - Boolean/Phrase | 2,548 | |
| S3 | ( ( (MH "Middle East Respiratory Syndrome Coronavirus") OR (MH "Middle East Respiratory Syndrome") ) ) OR ( TI ( (mers N5 (coronavirus* OR virus*)) OR mers-cov OR "middle east respiratory" OR "middle east virus" ) ) OR ( AB ( (mers N5 (coronavirus* OR virus*)) OR mers-cov OR "middle east respiratory" OR "middle east virus" ) ) | Search modes - Boolean/Phrase | 873 | |
| S2 | ( TI ( ((wuhan OR shanghai OR seafood OR beijing) AND (coronavirus* OR corona-virus* OR covid)) OR 2019-nCoV OR 2019nCoV OR nCoV2019 OR nCov-2019 OR COVID-19 OR COVID19 OR SARS-CoV-2 OR HCoV-19 ) OR TI ( (coronavirus* OR corona-virus* OR covid OR HCoV OR CoV OR betacoronavirus*) N3 (new OR novel OR "2019" OR Wuhan OR Hubei OR China OR Chinese) ) OR TI ((Wuhan OR Hubei) N5 (pneumonia*)) ) OR ( AB ( ((wuhan OR shanghai OR seafood OR beijing) AND (coronavirus* OR corona-virus* OR covid)) OR 2019-nCoV OR 2019nCoV OR nCoV2019 OR nCov-2019 OR COVID-19 OR COVID19 OR SARS-CoV-2 OR HCoV-19 ) OR AB ( (coronavirus* OR corona-virus* OR covid OR HCoV OR CoV OR betacoronavirus*) N3 (new OR novel OR "2019" OR Wuhan OR Hubei OR China OR Chinese) ) OR AB ((Wuhan OR Hubei) N5 (pneumonia*)) ) | Search modes - Boolean/Phrase | 1,191 | |
| S1 | ( (MH "Coronavirus Infections+") OR (MH "Coronavirus+") ) OR TI ( coronavirus* OR corona-virus* OR covid ) OR AB ( coronavirus* OR corona-virus* OR covid ) | Limiters - Published Date: 20190101-20211231 Search modes - Boolean/Phrase | 908 | |
